# Mistletoe in Cancer Cell Biology: Recent Advances

**DOI:** 10.3390/cimb47080672

**Published:** 2025-08-20

**Authors:** Chang-Eui Hong, Su-Yun Lyu

**Affiliations:** 1 College of Pharmacy, Sunchon National University, Suncheon 57922, Republic of Korea; 2 Smart Beautytech Research Institute, Sunchon National University, Suncheon 57922, Republic of Korea; 3 Research Institute of Life and Pharmaceutical Sciences, Sunchon National University, Suncheon 57922, Republic of Korea

**Keywords:** mistletoe, *Viscum album*, immunogenic cell death, macrophage polarization, 3D culture, cancer immunotherapy, PD-1/PD-L1 inhibitors, integrative oncology

## Abstract

Mistletoe (*Viscum album* L.) has been used in complementary cancer therapy for decades, but its mechanisms remained poorly understood until recently. This review synthesizes transformative advances in mistletoe cancer research from 2020 to 2025, focusing on newly discovered molecular mechanisms, immunomodulatory properties, and clinical applications. We conducted a comprehensive analysis of controlled studies, mechanistic investigations, and real-world evidence published between 2020 and 2025. The discovery of mistletoe-induced immunogenic cell death (ICD) represents a paradigm shift in understanding its anticancer effects. Mistletoe extracts trigger endoplasmic reticulum stress, leading to calreticulin exposure in 18–51% of cancer cells and a 7-fold increase in adenosine triphosphate (ATP) release. Three-dimensional culture models revealed enhanced macrophage reprogramming effects, with a 15.8% increase in pro-inflammatory interleukin (IL)-6 and a 26.4% reduction in immunosuppressive IL-10. Real-world evidence from over 400 non-small-cell lung cancer patients shows that combining mistletoe with programmed death-1/programmed death-ligand 1 (PD-1/PD-L1) inhibitors doubles median overall survival (6.8 to 13.8 months), with biomarker-selected populations experiencing up to a 91.2% reduction in death risk. The Johns Hopkins Phase I trial established intravenous administration safety at 600 mg three times weekly. Advanced analytical approaches including metabolomics, chronobiology, and machine learning are enabling precision medicine applications. These findings position mistletoe as a scientifically validated component of integrative oncology, bridging traditional medicine with evidence-based cancer care. Future research should focus on ferroptosis mechanisms, single-cell immune profiling, and standardized clinical protocols.

## 1. Introduction

Cancer remains a leading cause of mortality worldwide, with conventional therapies often limited by resistance, severe side effects, and impact on quality of life [1,2,3,4]. Natural products have historically provided important sources for cancer therapeutics, with over 60% of anticancer drugs in the market being derived from or inspired by natural compounds [5]. In the past decade, natural products have demonstrated unique advantages including high biocompatibility, low toxicity, fewer side effects, wide bioactivities, and large structural diversity, making them attractive candidates for drug development [6,7]. The continued relevance of natural products in oncology is evidenced by ongoing drug approvals and their role in providing novel mechanisms for treating various cancer types [8,9,10].

Interest in natural product-based cancer therapies notably accelerated during the COVID-19 pandemic, as healthcare systems worldwide faced unprecedented challenges and cancer patients sought immune-enhancing complementary treatments to support their compromised immune systems during this vulnerable period [11,12]. Among these sought-after immunomodulatory agents, mistletoe extracts gained particular attention due to their documented immune-enhancing properties and established safety profile in cancer care [13,14,15]. Among these natural products, mistletoe (*Viscum album* L.) extracts have emerged as one of the most widely used complementary cancer treatments in Europe, with up to 77% of oncological patients in German-speaking countries utilizing mistletoe preparations to reduce tumor- and treatment-related symptoms and improve health-related quality of life [16,17,18].

Mistletoe extracts have been used in complementary cancer therapy primarily in European countries since the 1920s, introduced by Rudolf Steiner as part of anthroposophic medicine [19]. The plant contains multiple bioactive compounds with distinct mechanisms of action. Mistletoe extracts are typically prepared from leaves, stems, and sometimes berries of *V. album*, using various extraction methods including aqueous extraction, fermentation processes (e.g., lactic acid fermentation), and ethanolic preparations with different concentrations (10–70%). The choice of extraction method, host tree species, and plant material significantly influences the final concentration of bioactive compounds. The three types of mistletoe lectins (ML-I, ML-II, ML-III, purified from *Viscum album* leaves harvested from poplar, apple, and oak trees using aqueous extraction and affinity chromatography) are ribosome-inactivating proteins that share structural homology with ricin but demonstrate selective cytotoxicity toward cancer cells. Viscotoxins are small basic polypeptides (46 amino acids) that disrupt cell membranes, while polysaccharides and alkaloids contribute to immunomodulatory effects [20,21,22]. While historically used as supportive care to improve quality of life (QoL), mistletoe therapy has evolved from traditional remedy to evidence-based treatment, with recent meta-analyses demonstrating significant improvements in quality of life and potential survival benefits [23,24,25,26]. In Germany, mistletoe extracts are available as approved drugs based on monographs of the German Federal Institute for Drugs and Medical Devices, and are practiced in integration with conventional medicine and cancer chemotherapy [27]. The widespread acceptance is evident as mistletoe products are approved for subcutaneous use in Germany, Switzerland, Austria, Korea, and many other countries [18,28]. Clinical implementation has advanced rapidly, with real-world evidence from Germany demonstrating that combining mistletoe with programmed death-1/programmed death-ligand 1 (PD-1/PD-L1) inhibitors nearly doubles survival time in advanced lung cancer patients, with median overall survival extending from 6.8 to 13.8 months [29].

The period from 2020 to 2025 has witnessed transformative discoveries in mistletoe research, driven by technological advances and deeper mechanistic understanding. Previous reviews have focused primarily on clinical outcomes or quality of life measures, but none have comprehensively analyzed the cellular and molecular advances that explain mistletoe’s therapeutic effects [30,31,32,33]. A significant advance in 2025 demonstrated that mistletoe extract induces immunogenic cell death (ICD) [34]. Working with breast cancer and melanoma cell lines, researchers discovered that mistletoe triggers calreticulin exposure on 18–51% of viable cancer cells, releases adenosine triphosphate (ATP) at levels 7-fold higher than controls, and promotes heat shock protein translocation—all hallmarks of immunogenic death that alert the immune system to attack remaining cancer cells [34]. This mechanism appears to operate through endoplasmic reticulum stress pathways, with mistletoe lectins acting as ribosome-inactivating proteins that trigger phosphorylation of eIF2α without the typical stress response completion [34,35]. The discovery of ICD induction provides a potential mechanistic link that may explain mistletoe’s clinical benefits observed over decades. Furthermore, comparative analysis reveals that Korean mistletoe (*V. album* var. *coloratum*) consistently demonstrates 2–3-fold higher cytotoxic activity compared to European varieties, with unique macrophage polarization effects increasing interleukin (IL)-6 by 15.8% in M1 macrophages while reducing IL-10 by 26.4% in M2 macrophages [36,37].

Advanced research methodologies have revolutionized mistletoe research, with three-dimensional (3D) spheroid cultures revealing drug interactions invisible in traditional two-dimensional (2D) models [36,38]. These 3D models better recapitulate the tumor microenvironment by mimicking strong cell-to-cell interactions and mass transfer limitations of metabolites, oxygen, and drugs, which 2D models fail to replicate [39,40]. Multi-omics approaches have mapped mistletoe’s complex molecular landscape with unprecedented detail, with metabolomics studies identifying 188+ distinct metabolites that vary based on host tree and preparation method [41]. Mass spectrometry-based proteomics discovered approximately 200 additional mitochondrial proteins in mistletoe-treated cells, suggesting profound metabolic reprogramming beyond simple cytotoxicity [42]. Clinical translation has accelerated with the Johns Hopkins Phase I trial establishing intravenous mistletoe’s maximum tolerated dose at 600 mg three times weekly, demonstrating 25% stable disease rate (patients showing neither tumor progression nor sufficient shrinkage to qualify as partial response) with median follow-up of 15.3 months [43]. Real-world data studies have shown that adding mistletoe therapy to PD-1/PD-L1 inhibitors in advanced non-small-cell lung cancer (NSCLC) patients is associated with a 56% reduction in the adjusted hazard of death (adjusted hazard ratio (aHR) 0.44, 95% confidence interval (CI): 0.26–0.74, *p* = 0.002), with some subgroups showing up to 75% reduction, all without increasing adverse events [29,44,45,46]. However, these observational data require confirmation through randomized controlled trials.

This review synthesizes the transformative advances in mistletoe cancer research from 2020 to 2025, examining how mistletoe transitioned from empirical use to evidence-based application. Unlike previous reviews that focused on clinical outcomes or anthroposophic perspectives, we analyze both the cellular and molecular advances and their clinical translation during this critical period, including the discovery of immunogenic cell death mechanisms, synergy with immunotherapy, and technological innovations in 3D culture systems and multi-omics approaches. By comprehensively analyzing mechanistic discoveries, controlled studies, real-world evidence, and emerging therapeutic strategies, this review demonstrates how molecular insights have enabled mistletoe’s evolution from traditional remedy to evidence-based therapy in modern oncology.

## 2. Cell Death Mechanisms: Recent Discoveries

**Figure 1:**
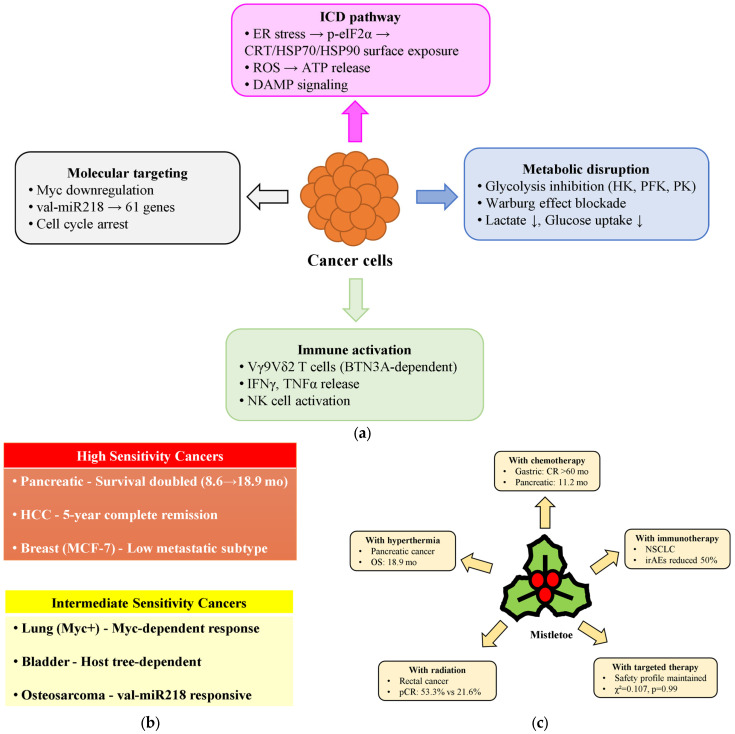
Molecular mechanisms and therapeutic applications of mistletoe in cancer treatment (2020–2025). (**a**) Four major pathways through which mistletoe affects cancer cells. (**b**) Stratification of cancer types by mistletoe sensitivity based on clinical responses. (**c**) Enhanced outcomes from combining mistletoe with conventional cancer therapies, showing specific benefits for each combination. BTN3A, butyrophilin 3A; CR, complete response; CRT, calreticulin; ER, endoplasmic reticulum; HCC, hepatocellular carcinoma; HK, hexokinase; HSP, heat shock protein; ICD, immunogenic cell death; irAEs, immune-related adverse events; NSCLC, non-small-cell lung cancer; OS, overall survival; pCR, pathologic complete response; PFK, phosphofructokinase; PK, pyruvate kinase.

This section examines the recent advances in understanding mistletoe’s anticancer mechanisms from 2020 to 2025, a period marked by significant discoveries in molecular targeting and clinical applications. We analyze three interconnected areas: (1) the molecular mechanisms underlying mistletoe’s anticancer effects, including immunogenic cell death induction, metabolic reprogramming, and novel microRNA (miRNA)-mediated regulation; (2) cell-type-specific responses revealing differential sensitivity patterns across cancer types; and (3) synergistic interactions with conventional therapies that enhance therapeutic outcomes while reducing toxicity. These advances, summarized in Table 1 and illustrated in Figure 1, demonstrate mistletoe’s evolution from traditional remedy to molecularly characterized therapeutic agent with defined mechanisms and predictable clinical responses.

### 2.1. Molecular Mechanisms of Action

European mistletoe extracts exert anticancer effects through multiple interconnected mechanisms. Weissenstein et al. (2025) demonstrated that mistletoe treatment triggers immunogenic cell death characterized by endoplasmic reticulum stress and surface exposure of damage-associated molecular patterns (DAMPs) [34]. Fermented *V. album* extract (VAE, prepared from leaves, stems and berries harvested from oak trees, ground and fermented for 3 days with *Lactobacillus plantarum* in aqueous solution) induces phosphorylation of eIF2α and increases surface expression of calreticulin, heat shock protein (HSP) 70, and HSP90 on early apoptotic cells, along with mitochondrial reactive oxygen species (ROS) production and ATP release [34]. The apoptotic cascade involves mitochondrial membrane depolarization, with mistletoe fruit extract showing higher depolarization rates than 5-fluorouracil in Ehrlich ascites tumor cells [54]. Caspase-3 activation represents a central execution mechanism, particularly evident in radiation-resistant rectal cancer cells where mistletoe treatment restored apoptotic sensitivity [51].

Mistletoe induces metabolic reprogramming by inhibiting key glycolytic enzymes—hexokinase, phosphofructokinase, and pyruvate kinase—effectively interrupting the Warburg effect [41]. This metabolic disruption was most pronounced with extracts from *Quercus* sp. and *Abies alba* host trees, demonstrating dose-dependent reduction in glucose uptake and lactate production in MDA-MB-231 breast cancer cells [41].

Beyond direct cytotoxicity, mistletoe extracts profoundly modulate immune surveillance. Non-fermented mistletoe extracts (AbnobaViscum, prepared from fresh mistletoe herb using aqueous extraction with disodium phosphate dihydrate, ascorbic acid, and water for injection, plant to extract ratio 1:50) induce specific expansion of Vγ9Vδ2 T cells through butyrophilin 3A (BTN3A)-dependent mechanisms, resulting in rapid cytotoxic granule release and production of interferon-gamma (IFN-γ) and tumor necrosis factor-alpha (TNF-α) [52]. In Myc-amplified small-cell lung cancer, ML (commercial preparation from Sigma-Aldrich, Saint Louis, MO, USA) specifically decreased expression of amplified C-myc and N-myc proteins, with overexpression of either myc variant paradoxically enhancing cellular sensitivity to treatment [50]. The recent discovery that mistletoe-derived val-miR218 targets 61 genes involved in cell cycle regulation introduces plant-derived miRNAs as novel therapeutic entities [48].

### 2.2. Cell-Type-Specific Responses and Therapeutic Windows

The therapeutic efficacy of mistletoe extracts varies dramatically across cancer types. Pancreatic cancer demonstrates notable responsiveness, with median survival extending from 8.6 months with chemotherapy alone to 11.2 months with added mistletoe, reaching 18.9 months when hyperthermia completed the triple combination [49]. The metabolic vulnerability of pancreatic tumors may underlie this sensitivity, as mistletoe has demonstrated broad metabolic benefits, including improved stress tolerance in experimental models where 40% ethanol extracts increased physical performance by 3.4–5.05 times [55].

Hepatocellular carcinoma (HCC) represents another highly sensitive malignancy. A case report documented complete remission of advanced multifocal HCC achieved with intravenous mistletoe and L-ornithine L-aspartate sustained for 5 years demonstrates the potential for durable responses [56]. Egyptian studies on 45 patients with intermediate HCC showed stable disease in all patients treated with VAE, accompanied by significant angiogenic modulation—increased vascular endothelial growth factor (VEGF) and TNF-α with decreased transforming growth factor-beta (TGF-β) [57]. Cell line studies revealed selective cytotoxicity, with VAD30 (1x10-30) decreasing HepG2 viability while sparing mesenchymal stem cells [47].

Breast cancer cells exhibit differential sensitivity correlating with metastatic potential. Low metastatic MCF-7 cells demonstrated greater susceptibility to Iscador^®^ (fermented aqueous mistletoe extract from oak or apple host trees, manufactured as 20 mg/mL solution for injection) than highly metastatic MDA-MB-231 cells, with selective alterations in membrane fluidity and zeta potential occurring only in MCF-7 [58]. Summer-harvested mother tinctures (ethanolic extracts from fresh *V. album* harvested in summer from various host trees including apple, oak, elm, fir, and pine, prepared using 21-day ethanolic maceration at 40–50% alcohol content) inhibited glycolytic enzymes most effectively in triple-negative breast cancer cells [41].

Lung cancer responses are stratified by their molecular characteristics. Small-cell lung cancer with Myc amplification showed exquisite sensitivity to mistletoe lectin, which specifically decreased expression of the amplified oncogene [50]. Non-small-cell lung cancer cells treated with plant-produced ML-II achieved half maximal effective concentration (EC50) values of 4 μg/mL (H460) and 3.5 μg/mL (A549) [59].

Bladder cancer demonstrates host tree-dependent responses, with Salicis and Populi extracts inducing the strongest growth inhibition through distinct mechanisms—Salicis downregulated cyclin-dependent kinase (CDK)1/2 and Cyclin A, while Populi affected only CDK2 and Cyclin A [60]. Novel opportunities emerge in osteosarcoma, which responded to mistletoe-derived val-miR218 targeting cell cycle genes [48], and medulloblastoma, where Daoy cells underwent caspase-mediated apoptosis at 0.05 mg/mL [61].

### 2.3. Synergistic Effects with Conventional Therapies

The integration of mistletoe extracts with conventional cancer therapies reveals synergistic interactions. In recurrent gastric cancer with peritoneal carcinomatosis, the combination of docetaxel and mistletoe achieved complete remission sustained beyond 60 months [53]. Advanced pancreatic cancer showed stepwise survival improvements: 8.6 months with chemotherapy alone, 11.2 months adding mistletoe, and 18.9 months with triple therapy including hyperthermia [49].

Radiation therapy synergies are evident in locally advanced rectal cancer, where patients receiving chemoradiotherapy with mistletoe achieved pathologic complete response rates of 53.3% versus 21.6% without mistletoe (*p* = 0.044) [51]. For lung cancer patients, radiation combined with mistletoe significantly improved symptom control, reducing pain scores by 27 points (*p* = 0.006) and nausea/vomiting by 17 points (*p* = 0.005) [62].

The safety of mistletoe with targeted therapies was confirmed in 242 breast and gynecological cancer patients, where combining mistletoe with poly (ADP-ribose) polymerase (PARP) inhibitors, CDK4/6 inhibitors, or monoclonal antibodies maintained identical safety profiles (χ^2^ = 0.107, *p* = 0.99) [63].

Hyperthermia combinations demonstrate particular promise. Modulated electro-hyperthermia with *V. album* var. *coloratum* (Korean mistletoe aqueous extract prepared from leaves, berries, and 1–4 year old stems collected from oak trees using distilled water extraction at 4 °C with repeated extraction cycles) triggered robust immunological responses, elevating cytotoxic T lymphocyte activity and increasing IFN-γ and granzyme secretion [64]. Clinical validation in pancreatic cancer demonstrated the triple combination achieving 18.9 months median survival—more than double chemotherapy alone [49].

Quality of life improvements are consistent across combination therapies. While chemotherapy, immunotherapy, and endocrine therapy caused 17, 17, and 6-point deteriorations in fatigue scores, respectively, concurrent mistletoe improved fatigue by 12 points (*p* = 0.0004) [65].

## 3. Immunomodulatory Properties: New Insights

### 3.1. Macrophage Reprogramming and TAM Modulation

Tumor-associated macrophages (TAMs) constitute up to 50% of tumor mass in some cancers, making them critical therapeutic targets. While conventional strategies focus on TAM depletion or recruitment blockade with limited clinical success [66,67,68], mistletoe offers a fundamentally different approach: reprogramming existing TAMs from pro-tumor M2 to anti-tumor M1 phenotypes without depleting beneficial tissue-resident macrophages.

**Figure 2:**
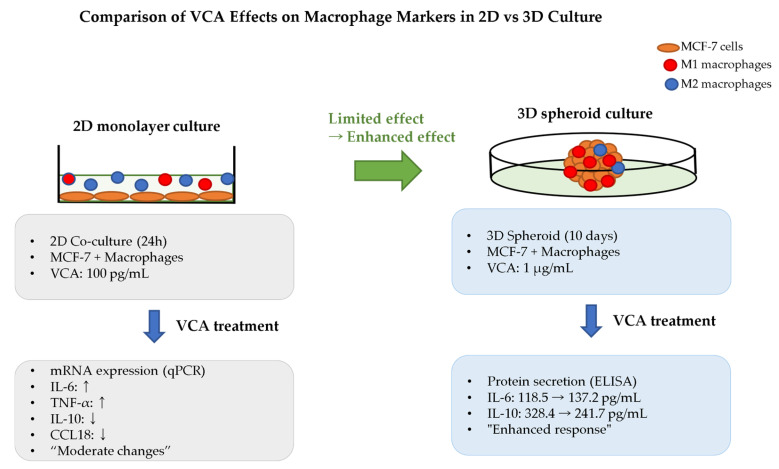
Comparison of VCA effects on macrophage polarization markers between 2D and 3D culture systems. Left panel shows 2D co-culture of MCF-7 cells with M1 or M2 macrophages treated with 100 pg/mL VCA for 24 h, measuring mRNA expression changes by qPCR. Right panel shows 3D spheroid co-culture treated with 1 μg/mL VCA for 10 days, measuring protein secretion by ELISA. The 3D culture system demonstrates enhanced cytokine modulation compared to 2D culture, with more pronounced changes in IL-6 (118.5 to 137.2 pg/mL in MCF-7+M1) and IL-10 (328.4 to 241.7 pg/mL in MCF-7+M2). Note the different measurement methods (mRNA vs. protein) and treatment conditions between the two systems. CCL18, C-C motif chemokine ligand 18; ELISA, enzyme-linked immunosorbent assay; IL, interleukin; M1, classically activated macrophage; M2, alternatively activated macrophage; qPCR, quantitative polymerase chain reaction; TNF-α, tumor necrosis factor-alpha; VCA, *Viscum album* var. *coloratum* agglutinin.

Hong and Lyu (2024) provided the first comprehensive evidence that *V. album* var. *coloratum* induces precise macrophage reprogramming in breast cancer systems [36]. Their quantitative analysis revealed bidirectional modulation using *V. album* var. *coloratum* agglutinin (VCA, Korean mistletoe lectin purified from air-dried plant material using aqueous extraction followed by SP Sephadex C-50 ion exchange and asialofetuin-Sepharose 4B affinity chromatography with acetate buffer). VCA increased pro-inflammatory IL-6 by 15.8% in M1 macrophages while reducing immunosuppressive IL-10 by 26.4% in M2 macrophages. Notably, these effects were significantly enhanced in 3D spheroid cultures compared to traditional 2D systems, suggesting that previous studies may have underestimated mistletoe’s immunomodulatory potential due to methodological limitations [36]. This finding has important implications for re-evaluating historical mistletoe research and optimizing future clinical applications. Figure 2 illustrates this dramatic enhancement of mistletoe’s immunomodulatory effects in 3D culture systems compared to traditional 2D models, demonstrating how three-dimensional tumor microenvironments are essential for capturing the full therapeutic potential of VCA.

The molecular mechanisms underlying mistletoe-induced TAM reprogramming involve coordinated multi-pathway activation that distinguishes it from conventional single-target approaches. Current TAM-targeting strategies include iron-based nanoparticles that induce M1 polarization through ROS generation [69] and miRNA-mediated approaches requiring complex delivery systems [68]. In contrast, mistletoe components simultaneously engage multiple pathways: toll-like receptor (TLR) 4 signaling mediates M2b regulatory phenotype emergence, as demonstrated in fever-range hyperthermia studies [70], while comprehensive cytokine reprogramming occurs in the tumor microenvironment. Specifically, *V. album* var. *coloratum* suppressed IL-6 secretion in co-cultures while elevating IL-4, TGF-β, and IFN-γ, creating an immune milieu unfavorable to tumor progression [71]. This multi-modal action may explain mistletoe’s favorable safety profile compared to targeted therapies.

The Korean variety shows distinct macrophage-modulating activity, correlating with structural differences in their lectins. The Korean variety’s unique mistletoe lectin induces mitochondria-mediated cancer cell apoptosis (increased Bcl-2-associated X protein (Bax)/B-cell lymphoma 2 (Bcl-2) ratios) while simultaneously activating macrophages through caspase-3-dependent mechanisms [71]. This dual action was amplified when combined with modulated electro-hyperthermia, which not only suppressed TAM differentiation signals but also enhanced cytotoxic T lymphocyte activity with increased IFN-γ and granzyme secretion in melanoma models [64]. Such synergistic effects between botanical and physical therapies represent innovative approaches to overcome immunologically “cold” tumors.

To determine whether macrophage modulation is a conserved property across mistletoe species, comparative studies provide valuable insights. *Passovia ovata*, a South American mistletoe, demonstrated anti-inflammatory activity at 500 μg/mL, inhibiting nitric oxide (NO) production and reducing IL-1β, IL-6, and TNF-α through TLR4 pathway modulation [72]. While these findings confirm anti-inflammatory properties across mistletoe genera, the specific ability to reprogram TAMs toward anti-tumor phenotypes appears unique to *V. album*, particularly the Korean variety [36,71]. This specificity likely reflects evolutionary adaptation to different host trees and geographical environments.

Mistletoe’s TAM reprogramming strategy aligns with emerging immunotherapy paradigms emphasizing immune normalization over aggressive intervention. Unlike TAM depletion strategies that risk losing beneficial macrophage functions [67] or exosome-based approaches facing manufacturing challenges [66], mistletoe offers a practical reprogramming solution. The mechanism is supported by broader evidence that dietary lectins modulate immunity through direct interaction with macrophages, dendritic cells, and lymphocytes [73]. This positions mistletoe as a bridge between innate and adaptive immunity, particularly relevant for combination with checkpoint inhibitors.

Critical questions remain regarding the durability and optimization of mistletoe-induced TAM reprogramming. The temporal stability of reprogrammed macrophages requires investigation, especially considering that tumor-derived factors like IL-17A can rapidly reverse M1 polarization [74]. Additionally, the optimal dosing regimen, route of administration, and patient selection criteria need clarification. As shown in Figure 2, the enhanced macrophage reprogramming observed in 3D cultures provides critical insights for optimizing clinical protocols. Hong and Lyu’s 3D culture breakthrough suggests that integrating advanced technologies like single-cell sequencing and spatial transcriptomics could identify macrophage subpopulations most responsive to mistletoe, enabling precision immunotherapy approaches. Understanding these dynamics will be crucial for translating mistletoe’s TAM reprogramming potential into improved clinical outcomes.

### 3.2. Modulation T Cell and NK Cell Activation

Mistletoe extracts from various species demonstrate significant immunomodulatory effects through the activation of T cells and natural killer (NK) cells, which are crucial effector cells in anti-tumor immunity. Non-fermented *V. album* extracts (AbnobaViscum) specifically stimulate and expand Vγ9Vδ2 T cells, inducing rapid release of cytotoxic granules and promoting the production of cytokines IFN-γ and TNF-α in a BTN3A-dependent manner [52]. This selective activation of Vγ9Vδ2 T cells represents a unique mechanism, as these cells possess MHC-independent recognition of tumor cells and potent killing potential [52].

The effects on conventional T cell populations are equally significant. In aged mice models, ethanol extract of *Dendrophthoe pentandra* (mango mistletoe) leaves (150–600 mg/kg) significantly increased CD4+ CD28+ T cells at doses of 300 and 600 mg/kg, and CD8+ CD28+ T cells at 600 mg/kg, with a strong correlation (r = 0.48) for IL-2 level increases [75]. Furthermore Helixor^®^ M (aqueous extract from fresh *Viscum album* grown on apple trees) administration has been associated with increased white blood cell counts and enhanced cytokine production, including IL-2 and interferon-gamma, which are essential for T lymphocyte activation and proliferation [43].

NK cell activation represents another critical mechanism of mistletoe’s immunomodulatory effects. A systematic review and meta-analysis by Cogo et al. (2023) identified that two randomized controlled trials (RCTs) specifically reported beneficial effects of mistletoe extracts on immune cells, with particular emphasis on natural killer cell enhancement in colorectal cancer patients [76]. The Phase I trial by Paller et al. further demonstrated that intravenous mistletoe administration upregulated NK cell cytotoxic activity markers and enhanced production of chemokines (C-X-C motif chemokine ligand (CXCL) 9, CXCL10) that recruit NK cells to tumor sites [43]. While these studies provide encouraging preliminary data, the systematic review noted that evidence remains limited by the small number of studies and methodological constraints [76].

Clinical evidence supports these in vitro findings. In a phase I trial of intravenous Helixor^®^ M in advanced cancer patients, treatment resulted in increased levels of CXCL9 and CXCL10, chemokines that mediate the recruitment of cytotoxic T and NK cells to solid tumors [43]. The combination of *V. album* extract (Korean mistletoe) with modulated electro-hyperthermia (mEHT) further enhanced these effects, eliciting cytotoxic T lymphocyte (CTL) immune responses and increasing IFN-γ and granzyme secretion in tumor-bearing mice [64].

The mechanisms underlying T cell activation appear to involve both direct and indirect pathways. *V. album* lectins can directly bind to T cell surface receptors, while the induction of heat shock proteins (HSPs) through combination therapies creates a more favorable microenvironment for immune response induction [64]. Additionally, intratumoral infiltration with CD8+ cells was significantly increased in colorectal cancer patients treated with *V. album* extracts combined with influenza vaccine, demonstrating enhanced T cell trafficking to tumor sites [77].

### 3.3. Dendritic Cell (DC) Maturation and Antigen Presentation

DCs serve as the critical bridge between mistletoe-induced tumor cell death and adaptive immune responses. As detailed in Section 2.1., mistletoe extracts trigger immunogenic cell death with DAMP release that can activate DCs [34]. However, direct studies examining DC responses to mistletoe remain remarkably limited in the post-2020 era.

The primary evidence for mistletoe’s effects on DCs comes from earlier work demonstrating that Korean mistletoe lectin B-chain (KML-B) functions as a TLR4 agonist, inducing robust upregulation of CD40, CD80, CD86, and MHC class II molecules on bone marrow-derived DCs, accompanied by enhanced secretion of T helper (Th) 1-promoting cytokines [78]. This direct DC activation complements the indirect effects mediated by mistletoe-activated lymphocytes (Section 3.2.), which produce IFN-γ—a potent inducer of DC maturation [52,79].

The therapeutic implications center on cross-presentation, whereby DCs present tumor antigens on MHC class I molecules to prime CD8+ T cells [80]. The combination of mistletoe-induced ICD providing tumor antigens and potential DC activation creates favorable conditions for anti-tumor immunity. Notably, mistletoe’s context-dependent immunomodulation—suppressing chronic inflammation while preserving acute responses necessary for DC function—may optimize this process [16].

Despite these mechanistic insights, systematic investigation of DC responses to mistletoe using contemporary approaches remains a critical gap, particularly regarding cross-presentation efficiency, DC subset-specific responses, and integration with checkpoint blockade therapies.

### 3.4. Cytokine Network Modulation

While previous sections examined mistletoe’s effects on specific immune cell populations, the modulation of cytokine networks represents a critical mechanism underlying mistletoe’s immunotherapeutic potential. Cytokines orchestrate the complex interplay between innate and adaptive immunity, with the balance between pro-inflammatory and anti-inflammatory mediators determining therapeutic outcomes in cancer [81].

Historically, mistletoe extracts have been shown to modulate various cytokines including TNF-α, IL-1, IL-6, and IFN-γ, though the specific patterns vary with preparation methods and cancer types [82]. However, comprehensive cytokine profiling studies using mistletoe in the post-2020 era remain remarkably limited. The Phase I trial by Paller et al. (2023) provided preliminary evidence of cytokine modulation, demonstrating increased levels of CXCL9 and CXCL10 following intravenous mistletoe administration in advanced cancer patients [43]. These chemokines are particularly relevant as they mediate the recruitment of cytotoxic T and NK cells to solid tumors. However, the authors emphasized that these cytokine measurements were “preliminary and hypothesis generating,” highlighting the need for more systematic investigation [43].

Recent meta-analyses have consistently noted mistletoe’s immunomodulatory effects and potential to reduce inflammatory markers, yet specific cytokine data remain sparse [83]. The context-dependent immunomodulation described by Nicoletti (2023)—suppressing chronic inflammatory mediators while preserving acute immune responses—suggests complex cytokine network effects that warrant detailed characterization [16]. The traditional classification of Th1/Th2 balance, the distinction between systemic and local cytokine effects, and the temporal dynamics of cytokine responses following mistletoe treatment all remain largely unexplored in contemporary studies.

Given the central role of cytokine networks in orchestrating anti-tumor immunity and the preliminary evidence of mistletoe’s modulatory effects, systematic profiling using modern multiplex technologies represents a critical research priority. Such studies should examine not only individual cytokine levels but also their functional networks, temporal dynamics, and correlation with clinical outcomes to fully elucidate mistletoe’s immunotherapeutic mechanisms.

The immunomodulatory effects of mistletoe on various immune cell populations from recent studies (2020–2025) are summarized in Table 2. These findings collectively demonstrate mistletoe’s multifaceted impact on both innate and adaptive immune responses, though significant gaps remain in our understanding of the integrated immune network effects.

## 4. Clinical Applications in the Modern Era

### 4.1. Breakthrough in Immunotherapy Combination

Building on the ICD mechanisms, real-world evidence now demonstrates remarkable clinical benefits when combining mistletoe with checkpoint inhibitors. Analysis of 300 patients with advanced NSCLC from the Network Oncology registry revealed that adding *V. album* to PD-1/PD-L1 inhibitor therapy doubled median overall survival from 6.8 to 13.8 months (*p* = 0.005), with three-year survival rates increasing from 8.0% to 16.5% [84]. A larger registry analysis of 405 patients confirmed these benefits, showing significantly higher three-year survival in the combination group (34.3% vs. 17.2%, *p* = 0.02) [44].

This synergy reflects complementary mechanisms identified in preclinical studies. The immunogenic cell death induced by chemotherapeutics such as cyclophosphamide and oxaliplatin, combined with *V. album*’s ability to enhance dendritic cell maturation and activate NK cells and macrophages, creates an optimal environment for checkpoint inhibitor efficacy [29]. Crucially, *V. album* does not interfere with PD-ligand expression on cancer cells, ensuring compatibility with checkpoint blockade mechanisms [85].

The Johns Hopkins Phase I trial provided critical validation, establishing intravenous mistletoe’s maximum tolerated dose at 600 mg three times weekly and documenting increased CXCL9 and CXCL10 levels—chemokines essential for T cell recruitment [43]. These findings, supported by systematic reviews of mistletoe’s immunomodulatory properties, confirm the translation from bench to bedside [44].

Safety data reinforces clinical feasibility across multiple studies. Among 242 breast and gynecological cancer patients, combining *V. album* with various targeted therapies maintained identical safety profiles (χ^2^ = 0.107, *p* = 0.99) [63]. In the larger lung cancer cohort, adverse event-related discontinuation was actually lower in the combination group (4.9% vs. 6.4%), with patients experiencing improved cancer-related fatigue and quality of life measures [65,86].

The most compelling evidence emerges from biomarker-selected populations. Among PD-L1-positive patients receiving first-line therapy, adding AbnobaViscum reduced death risk by 75% (aHR: 0.25; 95% CI: 0.11–0.60, *p* = 0.002) [84]. Notably, female NSCLC patients showed exceptional benefit, with a 91.2% reduction in death risk (aHR: 0.088; 95% CI: 0.009–0.783) [86]. These outcomes, achieved without additional toxicity, establish *V. album* as a valuable immunotherapy partner, bridging traditional medicine with cutting-edge oncology.

### 4.2. Integration with Conventional Therapies

The immunogenic cell death mechanisms elucidated in Section 2 provide scientific rationale for combining mistletoe with cytotoxic therapies. Across multiple cancer types, *V. album* demonstrates consistent benefits when integrated with standard treatments. A cost-effectiveness analysis revealed an incremental cost-effectiveness ratio of €3586 per life year gained, demonstrating economic viability alongside clinical benefits [87].

Standardized protocols are advancing through trials like MISTRAL in pancreatic cancer, which employs evidence-based dose escalation from 0.01 mg to 20 mg subcutaneously three times weekly alongside palliative chemotherapy, establishing methodological frameworks for integrative oncology [88]. For radiation combinations, lung cancer patients receiving concurrent *V. album* therapy experienced significant quality of life improvements, suggesting potential for mitigating treatment-related fatigue [62].

Beyond conventional cytotoxic combinations, innovative multimodal approaches are emerging. In pancreatic cancer, integrative protocols combining mistletoe with hyperthermia and high-dose vitamin C show promise for enhancing therapeutic outcomes [89]. Novel phytotherapeutic combinations, including mistletoe with curcumin and Boswellia, demonstrate potential synergistic effects warranting further investigation [90].

Consistent with the safety profile observed with targeted therapies (Section 4.1.), conventional therapy combinations maintain excellent tolerability. A systematic review of mistletoe in breast cancer patients confirmed improved quality of life across multiple trials without additional toxicity [24], while real-world data demonstrated enhanced treatment adherence trends [63]. These standardized protocols and emerging combination strategies provide the foundation for precision medicine applications discussed in the following section. The clinical outcomes of mistletoe combination therapies across different cancer types are summarized in Table 3, demonstrating consistent improvements in survival and response rates when mistletoe is integrated with conventional treatments.

### 4.3. Precision Medicine Applications

Building on the ICD mechanisms discovered by Weissenstein et al. [34] and the 3D model insights from Hong et al. [36] elucidated in Section 2, precision medicine applications for mistletoe therapy are rapidly evolving from empirical use to biomarker-guided approaches. The metabolomic profiling of mistletoe preparations reveals remarkable complexity, with 212 metabolites identified through comprehensive liquid chromatography-high resolution mass spectrometry/mass spectrometry (LC-HRMS/MS) analysis, showing clear separation between preparations from different host trees (Malus domestica vs. Pinus sylvestris) by principal component analysis [91]. This metabolomic fingerprinting provides a foundation for quality standardization and potentially predicting therapeutic responses based on preparation characteristics.

The discovery of mistletoe-induced ICD opens new avenues for patient stratification. As demonstrated with PD-L1 status in Section 4.1., the ICD markers—calreticulin exposure, ATP release, and high mobility group box 1 (HMGB1) secretion—may serve as predictive biomarkers for treatment response. Recent investigations into mistletoe’s interaction with survivin protein, an apoptosis inhibitor overexpressed in cancer, revealed that quercetin, rosmarinic acid, and catechin from mistletoe bind effectively to this target, suggesting potential for molecular-level patient selection [92].

Individual patient responses to mistletoe therapy demonstrate considerable heterogeneity across multiple dimensions. A qualitative study analyzing patient experiences identified six distinct levels of response: physical, vital, emotional, mental, spiritual, and social, with fever response emerging as a cross-dimensional phenomenon potentially serving as a personalized dosing marker [93]. This multidimensional response profile aligns with the principle that mistletoe therapy must be individualized according to each patient’s clinical case and treatment response [33].

Advances in analytical technologies enable sophisticated characterization of mistletoe preparations and responses. Chronobiological analysis spanning 27 years revealed characteristic self-correlation patterns (statistical measures showing how mistletoe’s metabolomic fingerprints correlate with themselves over time, with patterns repeating at regular intervals) of approximately 50 days, suggesting inherent rhythmic properties that could guide optimal dosing schedules [94]. Furthermore, machine learning approaches have successfully classified mistletoe preparations based on their fractal patterns, with unsupervised deep learning distinguishing between turbulent, laminar, and diffusion-based mixing procedures through 13 distinct clusters, demonstrating the potential for artificial intelligence (AI)-driven quality control and standardization [95].

These precision medicine tools—from metabolomic profiling and chronobiological patterns to machine learning classification—transform mistletoe therapy from traditional empiricism to data-driven personalization. The convergence of Weissenstein et al.’s [34] ICD discovery with Hong et al.’s [36] 3D model demonstration and these advanced analytical approaches establishes a framework for biomarker-guided protocols, setting the stage for the clinical translation strategies discussed in the following section.

## 5. Clinical Translation: From Europe to Global Implementation

The groundbreaking discoveries of mistletoe’s ICD mechanisms and 3D model validation have catalyzed a paradigm shift in its clinical application. This section traces the journey from traditional European use to emerging global acceptance, highlighting pivotal milestones in regulatory approval, real-world evidence integration, and standardization efforts that are transforming mistletoe from folk remedy to evidence-based therapy.

### 5.1. The Johns Hopkins Breakthrough

The landscape of mistletoe therapy in the United States fundamentally changed when Johns Hopkins University initiated the first Food and Drug Administration (FDA)-approved intravenous mistletoe trial, publishing results in 2023 [43]. This milestone occurred within evolving regulatory frameworks, as the FDA’s approach to botanical drug development had received over 700 investigational new drug applications, with cancer management representing the largest category [96].

The Johns Hopkins phase I trial enrolled 21 patients with various advanced cancers, establishing intravenous dosing at 600 mg three times weekly. Addressing the limitation of subcutaneous administration—which caused local pain and swelling—the intravenous protocol achieved 25% stable disease rate with median follow-up of 15.3 months. Mechanistically, elevated CXCL9, CXCL10, and granulocyte colony-stimulating factor (G-CSF) levels indicated immune activation capable of recruiting cytotoxic T and natural killer cells [43].

Contemporary reviews have contextualized these findings within decades of European experience. Klingemann’s 2024 analysis highlighted that historical trials by Tröger et al. in pancreatic cancer and others in hepatocellular carcinoma had established precedent, though these predated current evidence standards [97]. Similarly, systematic reviews by Biegel et al. (2022) documented mistletoe’s therapeutic potential in companion animals, opening new translational avenues [98].

Recent regulatory analyses confirm mistletoe’s unique position in botanical drug development. Among cancer-related botanical investigational new drugs (INDs) submitted to FDA through 2020, mistletoe preparations represented a significant proportion of active investigations, with several advancing to late-phase trials [96]. This regulatory momentum, combined with patient advocacy and the Johns Hopkins precedent, positions mistletoe at the forefront of integrative oncology’s evidence-based evolution in America.

### 5.2. Regulatory Evolution and Future Directions

The Johns Hopkins breakthrough catalyzed fundamental shifts in regulatory approaches to botanical cancer therapeutics. The FDA’s evolving framework, analyzed comprehensively by Wu et al. (2020), revealed that botanical drugs face unique challenges due to their complex mixtures and variable compositions influenced by cultivation practices, climate conditions, and processing protocols [99]. This complexity necessitated new regulatory paradigms beyond traditional single-molecule drug development.

Park et al.’s 2025 analysis of FDA botanical submissions demonstrated that among over 700 INDs received since 1984, cancer indications dominated, yet only two new drug applications (NDAs) for botanicals achieved approval, namely Veregen in 2006 and Fulyzaq in 2012 [96]. This stark disparity between submissions and approvals highlighted systemic challenges that mistletoe’s advancement could help address. Notably, 69% of oncologic botanical INDs received initial safe-to-proceed designations compared to 58% for non-oncologic indications, suggesting regulatory receptiveness to cancer applications [96].

The regulatory landscape evolved significantly with the 2016 revised Botanical Drug Development Guidance, which addressed critical late-phase trial considerations [99]. This guidance acknowledged that botanical products’ therapeutic consistency depends not only on chemical standardization but also on maintaining relationships between multiple active compounds—a principle particularly relevant to mistletoe’s immunomodulatory mechanisms involving multiple lectins and viscotoxins.

Contemporary regulatory science has embraced adaptive trial designs accommodating botanical medicines’ unique characteristics. The COVID-19 pandemic accelerated regulatory innovation, as documented by Gravelin et al. (2021), where Clinical and Translational Science Awards (CTSA) institutes facilitated expanded access programs that could serve as models for botanical drug development [100]. These frameworks enable real-world evidence collection while maintaining safety oversight—crucial for therapies like mistletoe with extensive traditional use but limited formal trial data.

Future regulatory directions point toward integrated evidence frameworks combining traditional use data, mechanistic studies, and pragmatic trials. The implication extends beyond individual products: Park et al. (2025) identified over 80 currently active botanical oncologic INDs, with several advancing to phase 3 trials, demonstrating viable pathways for botanical drug development in oncology despite historical challenges [96].

## 6. Conclusions

The period from 2020 to 2025 has transformed our understanding of mistletoe in cancer therapy, establishing molecular mechanisms that validate decades of empirical use. Recent molecular validations including enhanced efficacy in 3D culture systems [36] provide the scientific foundation for mistletoe’s therapeutic effects. These advances have revolutionized our approach to botanical cancer therapeutics.

**Figure 3:**
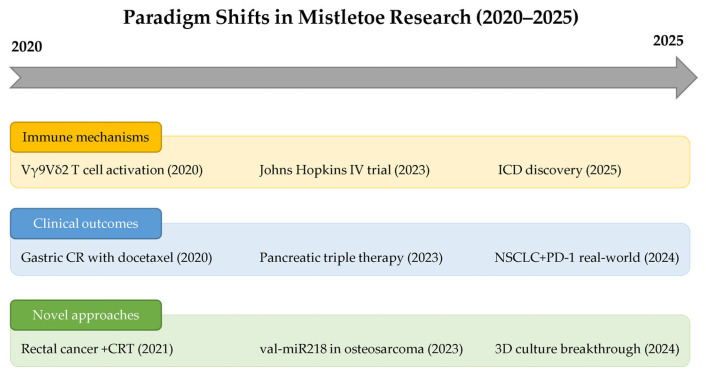
Paradigm shifts in mistletoe cancer research (2020–2025). Timeline highlighting transformative advances across three key areas: immune mechanisms, clinical outcomes, and novel therapeutic approaches. Major breakthroughs include γδ T cell activation mechanisms (2020), FDA-approved intravenous administration protocols (2023), and discovery of immunogenic cell death pathways (2025). The progression demonstrates evolution from initial mechanistic discoveries to clinical translation and innovative applications, culminating in enhanced 3D culture models (2024) and real-world evidence of immunotherapy synergy (2024). CR, complete response; CRT, chemoradiotherapy; ICD, immunogenic cell death; IV, intravenous; NSCLC, non-small-cell lung cancer; PD-1, programmed death-1.

Clinical translation has yielded compelling real-world evidence. The integration of mistletoe with checkpoint inhibitors has demonstrated favorable clinical outcomes [29,44,84], while maintaining excellent safety profiles across diverse combination therapies [63,86]. Figure 3 summarizes these transformative advances from 2020 to 2025, illustrating the progression from mechanistic discoveries to clinical implementation and novel therapeutic approaches. The Johns Hopkins trial’s establishment of intravenous protocols [43] marks a regulatory milestone, paving the way for broader implementation in Western oncology.

Looking forward, several critical areas warrant investigation. Ferroptosis mechanisms remain unexplored despite emerging evidence of metabolic reprogramming [41]. Single-cell sequencing technologies could elucidate the heterogeneous immune responses observed clinically. The development of AI-driven standardization protocols [95] and biomarker-guided patient selection strategies will be essential for optimizing therapeutic outcomes. Additionally, the One Health approach, leveraging cross-species applications [98], offers unique opportunities for accelerated drug development.

As precision oncology evolves, mistletoe therapy exemplifies the successful integration of traditional medicine with cutting-edge science. The convergence of multi-omics profiling [91], advanced culture models, and real-world evidence positions mistletoe as a valuable component of future cancer care. By bridging complementary and conventional approaches, mistletoe therapy offers new therapeutic possibilities for improving patient outcomes while maintaining quality of life—fulfilling the ultimate goal of integrative oncology.

## Figures and Tables

**Table 1:** Major discoveries in mistletoe cancer research (2020–2025). Studies are arranged chronologically and include molecular mechanisms, cell-type-specific responses, and synergistic effects with conventional therapies. BTN3A, butyrophilin 3A; CRT, calreticulin; DAMP, damage-associated molecular pattern; ER, endoplasmic reticulum; HSP70/90, heat shock protein 70/90; HT, hyperthermia; ICD, immunogenic cell death; ML, mistletoe lectin; MSC, mesenchymal stem cell; pCR, pathologic complete response; p-eIF2α, phosphorylated eukaryotic initiation factor 2α; SCLC, small-cell lung cancer; VAD30, *Viscum album* D30 (homeopathic dilution 1x10-30); VAE, *Viscum album* extract; val-miR218, *Viscum album*-derived microRNA 218.

Year	Discovery	Cancer Type/Model	Key Finding	Mechanism	Ref.
2025	First evidence of mistletoe-induced ICD markers	Breast (SKBR3, MDA-MB-231, MCF-7), melanoma (B16F10)	ER stress (p-eIF2α↑), DAMPs (CRT, HSP70, HSP90), ROS↑, ATP release	Fermented VAE triggers ER stress-mediated ICD	[34]
2024	VAD30 shows selective cytotoxicity	HepG2 vs. MSCs	Decreased HepG2 viability, no MSC damage	Selective cancer cell targeting	[47]
2023	Plant miRNA inhibits osteosarcoma	Osteosarcoma	val-miR218 targets 61 cell cycle genes	Cross-kingdom miRNA regulation	[48]
	Triple therapy extends pancreatic cancer survival	Pancreatic cancer (*n* = 206)	OS: chemo 8.6 mo → +ML 11.2 mo → +ML+HT 18.9 mo	Multi-modal synergy	[49]
2022	ML targets Myc-amplified SCLC	Small-cell lung cancer	Myc overexpression enhances sensitivity	Direct Myc protein downregulation	[50]
2021	Mistletoe enhances chemoradiotherapy response	Rectal cancer (*n* = 52)	pCR: 53.3% vs. 21.6% (*p* = 0.044)	Enhanced apoptosis, caspase-3 activation	[51]
2020	Non-fermented mistletoe activates Vγ9Vδ2 T cells	Human T cells	AbnobaViscum induces specific expansion, IFNγ/TNFα production	BTN3A-dependent, phosphoantigen-independent	[52]
	Docetaxel + mistletoe achieves complete remission	Recurrent gastric cancer	Complete response sustained >60 months	Synergistic cytotoxicity + immune modulation	[53]

**Table 2:** Summary of mistletoe (*Viscum album* L.)’s effects on immune cell populations from recent studies (2020–2025). BTN3A, butyrophilin 3A; CTL, cytotoxic T lymphocyte; ICD, immunogenic cell death; IFN-γ, interferon-gamma; IL, interleukin; mEHT, modulated electro-hyperthermia; M1/M2, macrophage polarization states; TAM, tumor-associated macrophage; TNF-α, tumor necrosis factor-alpha.

Immune Cell Type	Effects Observed	Key Findings	Reference
Macrophages (TAMs)	M2→M1 reprogramming	-IL-6↑ 15.8% in M1 -IL-10↓ 26.4% in M2 -Enhanced in 3D cultures	[36]
γδ T cells	Activation and expansion	-Vγ9Vδ2 T cell expansion -IFN-γ and TNF-α production -BTN3A-dependent	[52]
CTLs	Enhanced responses	-Increased with mEHT -IFN-γ and granzyme↑	[64]
Dendritic cells	Limited direct data	-ICD provides maturation signals -Recent direct studies lacking	[34]
Cytokine network	Preliminary modulation	-CXCL9, CXCL10↑ -Comprehensive profiling needed	[43]

**Table 3:** Clinical outcomes of mistletoe combination therapies (2020–2025). Summary of key clinical studies demonstrating the efficacy of *Viscum album* extracts in combination with conventional cancer therapies. Data represent median overall survival times or response rates comparing combination therapy versus conventional therapy alone. The pancreatic cancer study showed stepwise improvements with sequential addition of treatments. CR, complete remission; CRT, chemoradiotherapy; HT, hyperthermia; NSCLC, non-small-cell lung cancer; OS, overall survival; pCR, pathologic complete response; PD-1/PD-L1, programmed death-1/programmed death-ligand 1; VA, *Viscum album*.

Cancer Type	Combination Therapy	N	Key Outcome	Reference
NSCLC	VA + PD-1/PD-L1 inhibitors	300	Mistletoe + PD-1	[84]
Pancreatic	VA + Chemo → +HT	206	OS: 8.6→11.2→18.9 months	[49]
Rectal	VA + CRT	52	pCR: 21.6%→53.3% (*p* = 0.044)	[51]
Gastric	VA + Docetaxel	1	CR sustained >60 months	[53]

## References

[1] Park E.H., Jung K.W., Park N.J., Kang M.J., Yun E.H., Kim H.J., Kim J.E., Kong H.J., Im J.S., Seo H.G. (2024). Cancer Statistics in Korea: Incidence, Mortality, Survival, and Prevalence in 2021. Cancer Res. Treat..

[2] Kang M.J., Jung K.W., Bang S.H., Choi S.H., Park E.H., Yun E.H., Kim H.J., Kong H.J., Im J.S., Seo H.G. (2023). Cancer Statistics in Korea: Incidence, Mortality, Survival, and Prevalence in 2020. Cancer Res. Treat..

[3] Market M., Tennakoon G., Auer R.C. (2021). Postoperative Natural Killer Cell Dysfunction: The Prime Suspect in the Case of Metastasis Following Curative Cancer Surgery. Int. J. Mol. Sci..

[4] Gatenby R.A., Silva A.S., Gillies R.J., Frieden B.R. (2009). Adaptive therapy. Cancer Res..

[5] Talib W.H., Daoud S., Mahmod A.I., Hamed R.A., Awajan D., Abuarab S.F., Odeh L.H., Khater S., Al Kury L.T. (2022). Plants as a Source of Anticancer Agents: From Bench to Bedside. Molecules.

[6] Zhang C., Xie H., Zhang Z., Wen B., Cao H., Bai Y., Che Q., Guo J., Su Z. (2022). Applications and Biocompatibility of Mesoporous Silica Nanocarriers in the Field of Medicine. Front. Pharmacol..

[7] Abrantes D.C., Rogerio C.B., de Oliveira J.L., Campos E.V.R., de Araújo D.R., Pampana L.C., Duarte M.J., Valadares G.F., Fraceto L.F. (2021). Development of a Mosquito Repellent Formulation Based on Nanostructured Lipid Carriers. Front. Pharmacol..

[8] Mathur S., Hoskins C. (2017). Drug development: Lessons from nature. Biomed. Rep..

[9] Corroon J., Kight R. (2018). Regulatory Status of Cannabidiol in the United States: A Perspective. Cannabis Cannabinoid Res..

[10] Izzo A.A., Stefanska B. (2025). Natural products and cancer: From drug discovery to prevention and therapy. Br. J. Pharmacol..

[11] Karaman Özlü Z., Klinç T., Özlü İ., Ünal H., Toraman R.L. (2022). The relationship between individuals’ use of complementary and alternative medicine during the pandemic in Turkey and their attitudes towards perceived COVID-19 risk. Eur. J. Integr. Med..

[12] Hijazi M.A., Shatila H., Abu Qiyas S., Aboul-Ela M., El-Lakany A., Naja F. (2023). Complementary and alternative medicine use during the COVID-19 pandemic: Community pharmacists’ knowledge, attitudes, and practices. Res. Social. Adm. Pharm..

[13] Graetz D.E., Sniderman E., Villegas C.A., Kaye E.C., Ragab I., Laptsevich A., Maliti B., Naidu G., Huang H., Gassant P.Y. (2022). Resilient health care in global pediatric oncology during the COVID-19 pandemic. Cancer.

[14] Eliyas S., Gressel O., Ben-Arye E., Vagedes J., Samuels N., Kassem S. (2024). Coming out of the Integrative Oncology Comfort Zone: Addressing Healthcare Providers’ Wartime-Related Concerns. Psychooncology.

[15] Lim K.H.J., Murali K., Thorne E., Punie K., Kamposioras K., Oing C., O’Connor M., Élez E., Amaral T., Garrido P. (2022). The impact of COVID-19 on oncology professionals-one year on: Lessons learned from the ESMO Resilience Task Force survey series. ESMO Open.

[16] Nicoletti M. (2023). The Anti-Inflammatory Activity of Viscum album. Plants.

[17] Weissenstein U., Kunz M., Urech K., Regueiro U., Baumgartner S. (2016). Interaction of a standardized mistletoe (*Viscum album*) preparation with antitumor effects of Trastuzumab in vitro. BMC Complement. Altern. Med..

[18] Melzer J., Iten F., Hostanska K., Saller R. (2009). Efficacy and safety of mistletoe preparations (*Viscum album*) for patients with cancer diseases. A systematic review. Forsch. Komplementmed..

[19] Legnani W. (2008). Mistletoe in conventional oncological practice: Exemplary cases. Integr. Cancer Ther..

[20] Duong Van Huyen J.P., Delignat S., Kazatchkine M.D., Kaveri S.V. (2003). Comparative study of the sensitivity of lymphoblastoid and transformed monocytic cell lines to the cytotoxic effects of Viscum album extracts of different origin. Chemotherapy.

[21] Huguet Soler M., Stoeva S., Schwamborn C., Wilhelm S., Stiefel T., Voelter W. (1996). Complete amino acid sequence of the A chain of mistletoe lectin I. FEBS Lett..

[22] Frantz M., Jung M.L., Ribereau-Gayon G., Anton R. (2000). Modulation of mistletoe (*Viscum album* L.) lectins cytotoxicity by carbohydrates and serum glycoproteins. Arzneimittelforschung.

[23] Loef M., Walach H. (2020). Quality of life in cancer patients treated with mistletoe: A systematic review and meta-analysis. BMC Complement. Med. Ther..

[24] Loef M., Paepke D., Walach H. (2023). Quality of Life in Breast Cancer Patients Treated With Mistletoe Extracts: A Systematic Review and Meta-Analysis. Integr. Cancer Ther..

[25] Ostermann T., Appelbaum S., Poier D., Boehm K., Raak C., Büssing A. (2020). A Systematic Review and Meta-Analysis on the Survival of Cancer Patients Treated with a Fermented *Viscum album* L. Extract (Iscador): An Update of Findings. Complement. Med. Res..

[26] Loef M., Walach H. (2022). Survival of Cancer Patients Treated with Non-Fermented Mistletoe Extract: A Systematic Review and Meta-Analysis. Integr. Cancer Ther..

[27] Vanhaverbeke C., Touboul D., Elie N., Prévost M., Meunier C., Michelland S., Cunin V., Ma L., Vermijlen D., Delporte C. (2021). Untargeted metabolomics approach to discriminate mistletoe commercial products. Sci. Rep..

[28] Lederer A.K., Rieger S., Schink M., Huber R. (2024). Pharmakokinetics of Mistletoe Lectins after Intravenous Application of a Mistletoe Product in Healthy Subjects. Pharmaceuticals.

[29] Schad F., Thronicke A., Hofheinz R.D., Matthes H., Grah C. (2024). Patients with Advanced or Metastasised Non-Small-Cell Lung Cancer with Viscum album L. Therapy in Addition to PD-1/PD-L1 Blockade: A Real-World Data Study. Cancers.

[30] Bryant S., Duncan L., Feder G., Huntley A.L. (2022). A pilot study of the mistletoe and breast cancer (MAB) trial: A protocol for a randomised double-blind controlled trial. Pilot. Feasibility Stud..

[31] Vella R., Giardino A., Pizzocaro E., Frigerio I., Bannone E., Vieni S., Butturini G. (2025). Unconventional Treatments for Pancreatic Cancer: A Systematic Review. Cancers.

[32] Staupe H., Buentzel J., Keinki C., Buentzel J., Huebner J. (2023). Systematic analysis of mistletoe prescriptions in clinical studies. J. Cancer Res. Clin. Oncol..

[33] Matthes H., Thronicke A., Hofheinz R.D., Baars E., Martin D., Huber R., Breitkreuz T., Bar-Sela G., Galun D., Schad F. (2020). Statement to an Insufficient Systematic Review on *Viscum album* L. Therapy. Evid. Based Complement. Alternat Med..

[34] Weissenstein U., Tschumi S., Leonhard B., Baumgartner S. (2025). A fermented Mistletoe (*Viscum album* L.) extract elicits markers characteristic for immunogenic cell death driven by endoplasmic reticulum stress in vitro. BMC Complement. Med. Ther..

[35] Beztsinna N., de Matos M.B.C., Walther J., Heyder C., Hildebrandt E., Leneweit G., Mastrobattista E., Kok R.J. (2018). Quantitative analysis of receptor-mediated uptake and pro-apoptotic activity of mistletoe lectin-1 by high content imaging. Sci. Rep..

[36] Hong C.E., Lyu S.Y. (2024). Modulation of Breast Cancer Cell Apoptosis and Macrophage Polarization by Mistletoe Lectin in 2D and 3D Models. Int. J. Mol. Sci..

[37] Lyu S.Y., Park S.M., Choung B.Y., Park W.B. (2000). Comparative study of Korean (Viscum album var. coloratum) and European mistletoes (*Viscum album*). Arch. Pharm. Res..

[38] Lyu S.Y., Meshesha S.M., Hong C.E. (2025). Synergistic Effects of Mistletoe Lectin and Cisplatin on Triple-Negative Breast Cancer Cells: Insights from 2D and 3D In Vitro Models. Int. J. Mol. Sci..

[39] Habanjar O., Diab-Assaf M., Caldefie-Chezet F., Delort L. (2021). 3D Cell Culture Systems: Tumor Application, Advantages, and Disadvantages. Int. J. Mol. Sci..

[40] Rodrigues D.B., Reis R.L., Pirraco R.P. (2024). Modelling the complex nature of the tumor microenvironment: 3D tumor spheroids as an evolving tool. J. Biomed. Sci..

[41] Melo M.N.O., Ochioni A.C., Zancan P., Oliveira A.P., Grazi M., Garrett R., Holandino C., Baumgartner S. (2022). Viscum album mother tinctures: Harvest conditions and host trees influence the plant metabolome and the glycolytic pathway of breast cancer cells. Front. Pharmacol..

[42] Schröder L., Hohnjec N., Senkler M., Senkler J., Küster H., Braun H.P. (2022). The gene space of European mistletoe (Viscum album). Plant J..

[43] Paller C.J., Wang L., Fu W., Kumar R., Durham J.N., Azad N.S., Laheru D.A., Browner I., Kachhap S.K., Boyapati K. (2023). Phase I Trial of Intravenous Mistletoe Extract in Advanced Cancer. Cancer Res. Commun..

[44] Thronicke A., Schad F., Debus M., Grabowski J., Soldner G. (2022). Viscum album L. Therapy in Oncology: An Update on Current Evidence. Complement. Med. Res..

[45] Schad F., Thronicke A., Steele M.L., Merkle A., Matthes B., Grah C., Matthes H. (2018). Overall survival of stage IV non-small cell lung cancer patients treated with Viscum album L. in addition to chemotherapy, a real-world observational multicenter analysis. PLoS ONE.

[46] Thronicke A., Steele M.L., Grah C., Matthes B., Schad F. (2017). Clinical safety of combined therapy of immune checkpoint inhibitors and Viscum album L. therapy in patients with advanced or metastatic cancer. BMC Complement. Altern. Med..

[47] Valle A.C.V., de Carvalho A.C., Rahme S.W., Araujo A.d.R.B., Malard P.F., Sena Brunel H.S. (2024). Comparison of cytotoxicity caused by Viscum album in human mesenchymal stem cells and hepatocellular carcinoma cells. Med. Res. Arch..

[48] Xie W., Delebinski C., Gürgen D., Schröder M., Seifert G., Melzig M.F. (2023). Inhibition of osteosarcoma by European Mistletoe derived val-miR218. Extracell. Vesicles Circ. Nucl. Acids.

[49] Hohneck A.L., Sadikaj L., Heinemann L., Schroeder M., Riess H., Gerhards A., Burkholder I., Heckel-Reusser S., Gottfried J., Hofheinz R.D. (2023). Patients with Advanced Pancreatic Cancer Treated with Mistletoe and Hyperthermia in Addition to Palliative Chemotherapy: A Retrospective Single-Center Analysis. Cancers.

[50] Shatat M.A., Gauthier B., Yoon S., Yuan E., Yang P., Narla G., Dowlati A., Lee R.T. (2023). Mistletoe lectin inhibits growth of Myc-amplified small-cell lung cancer. Cancer Med..

[51] Baek J.H., Jeon Y., Han K.W., Jung D.H., Kim K.O. (2021). Effect of mistletoe extract on tumor response in neoadjuvant chemoradiotherapy for rectal cancer: A cohort study. World J. Surg. Oncol..

[52] Ma L., Phalke S., Stévigny C., Souard F., Vermijlen D. (2020). Mistletoe-Extract Drugs Stimulate Anti-Cancer Vγ9Vδ2 T Cells. Cells.

[53] Oh S.J. (2020). Reducing Malignant Ascites and Long-Term Survival in a Patient with Recurrent Gastric Cancer Treated with a Combination of Docetaxel and Mistletoe Extract. Case Rep. Oncol..

[54] Ateş Ş., Ulger H., Yılmaz S., Şeker Karatoprak G., al Ö., Uçar S., Taştan M., Tokpinar A., Alpa Ş., Farooqi A. (2022). Evaluation of antitumoral effect of mistletoe fruit extract on Ehrlich ascites tumor cells with muse cell analyzer and argyrophilic nucleolar organizer region staining method. Postępy Hig. Med. Dośw..

[55] Pozdnyakov D., Adzhiahmetova S., Chervonnaya N., Voronkov A., Oganesyan E. (2021). Some aspects of the adaptogenic potential of European mistletoe (*Viscum album* L.) extracts under variable physical performance. J. Med. Plants.

[56] Orange M., Poidimani N., Crosignani A., Werthmann P.G., Bertotto C. (2022). Complete, Durable Remission of Advanced Hepatocellular Carcinoma under Treatment with Viscum album Extracts: A Case Report. Complement. Med. Res..

[57] Elmelegy M., Abdella H., El-Bakly W., Tolba M., El-Demerdash E. (2021). Effect of Viscum album Extract on Angiogenesis Mediators and Cytokines in Egyptian Patients with Intermediate Hepatocellular Carcinoma. Arch. Pharm. Sci. Ain Shams Univ..

[58] Robev B., Iliev I., Tsoneva I., Momchilova A., Nesheva A., Kostadinova A., Staneva G., Nikolova B. (2023). Antitumor Effect of Iscador on Breast Cancer Cell Lines with Different Metastatic Potential. Int. J. Mol. Sci..

[59] Mazalovska M., Kouokam J.C. (2020). Transiently Expressed Mistletoe Lectin II in Nicotiana benthamiana Demonstrates Anticancer Activity In Vitro. Molecules.

[60] Juengel E., Rutz J., Meiborg M., Markowitsch S.D., Maxeiner S., Grein T., Thomas A., Chun F.K., Haferkamp A., Tsaur I. (2023). Mistletoe Extracts from Different Host Trees Disparately Inhibit Bladder Cancer Cell Growth and Proliferation. Cancers.

[61] Menke K., Schwermer M., Eisenbraun J., Schramm A., Zuzak T.J. (2021). Anticancer Effects of Viscum album Fraxini Extract on Medulloblastoma Cells in vitro. Complement. Med. Res..

[62] Schad F., Steinmann D., Oei S.L., Thronicke A., Grah C. (2023). Evaluation of quality of life in lung cancer patients receiving radiation and *Viscum album* L.: A real-world data study. Radiat. Oncol..

[63] Schad F., Thronicke A. (2023). Safety of Combined Targeted and Helixor^®^
*Viscum album* L. Therapy in Breast and Gynecological Cancer Patients, a Real-World Data Study. Int. J. Environ. Res. Public Health.

[64] Kim Y., Hur J., Hong S.C., Jung J., Park C.H., Park J.B., Yoon T.J., Kim J.B., Yang S.H. (2025). Modulated electro-hyperthermia therapy combined with Korean mistletoe extract treatment exerts a strong anti-tumor activity by enhancing cellular and humoral immune responses in mice. Anim. Cells Syst..

[65] Oei S.L., Thronicke A., Kröz M., von Trott P., Schad F., Matthes H. (2020). Impact of Oncological Therapy and *Viscum album* L. Treatment on Cancer-Related Fatigue and Internal Coherence in Nonmetastasized Breast Cancer Patients. Integr. Cancer Ther..

[66] Abe C., Bhaswant M., Miyazawa T., Miyazawa T. (2023). The Potential Use of Exosomes in Anti-Cancer Effect Induced by Polarized Macrophages. Pharmaceutics.

[67] Xu C., Chen J., Tan M., Tan Q. (2025). The role of macrophage polarization in ovarian cancer: From molecular mechanism to therapeutic potentials. Front. Immunol..

[68] Wang C., Wang X., Zhang D., Sun X., Wu Y., Wang J., Li Q., Jiang G. (2023). The macrophage polarization by miRNAs and its potential role in the treatment of tumor and inflammation (Review). Oncol. Rep..

[69] Ding H., Zhang Y., Mao Y., Li Y., Shen Y., Sheng J., Gu N. (2023). Modulation of macrophage polarization by iron-based nanoparticles. Med. Rev..

[70] Kozłowski H.M., Sobocińska J., Jędrzejewski T., Maciejewski B., Dzialuk A., Wrotek S. (2023). Fever-Range Hyperthermia Promotes Macrophage Polarization towards Regulatory Phenotype M2b. Int. J. Mol. Sci..

[71] Lim W.-T., Hong C.-E., Lyu S.-Y. (2023). Immuno-Modulatory Effects of Korean Mistletoe in MDA-MB-231 Breast Cancer Cells and THP-1 Macrophages. Sci. Pharm..

[72] Magalhães I.F.B., Figueirêdo A.L.M., da Silva E.M., de Miranda A.A.B., da Rocha C.Q., da Silva Calabrese K., Almeida-Souza F., Abreu-Silva A.L. (2023). Effects of Passovia ovata Mistletoe on Pro-Inflammatory Markers In Vitro and In Vivo. Plants.

[73] Konozy E.H.E., Osman M.E.M. (2024). From inflammation to immune regulation: The dual nature of dietary lectins in health and disease. Heliyon.

[74] Bian Z., Wu X., Chen Q., Gao Q., Xue X., Wang Y. (2024). Oct4 activates IL-17A to orchestrate M2 macrophage polarization and cervical cancer metastasis. Cancer Immunol. Immunother..

[75] Kalim H., Pratama M.Z., Sermoati I., Yuniati M., Haryati N., Norahmawati E., Endharti A., Irwanto Y., Solikhin M., Hidayat S. (2021). The Effect of Mango Mistletoes (*Dendrophthoe pentandra*) Leaves Extract on Percentage of CD4+CD28+, CD8+CD28+, and interleukin-2 Levels of Aged Balb/c Mice. Open Access Maced. J. Med. Sci..

[76] Cogo E., Elsayed M., Bhardwaj S., Cooley K., Aycho C., Liang V., Papadogianis P., Psihogios A., Seely D. (2023). Mistletoe Extracts during the Oncological Perioperative Period: A Systematic Review and Meta-Analysis of Human Randomized Controlled Trials. Curr. Oncol..

[77] Kaesbach S., Hintze A., Engelbrecht S., Wartenberg M., Templeton A.J. (2025). ER+ HER2- Invasive Breast Cancer: Tumor Remission following Viscum Album Extract/Influenza Vaccine Treatment—A Report of 2 Cases. Complement. Med. Res..

[78] Kim J.J., Hwang Y.H., Kang K.Y., Kim I., Kim J.B., Park J.H., Yoo Y.C., Yee S.T. (2014). Enhanced dendritic cell maturation by the B-chain of Korean mistletoe lectin (KML-B), a novel TLR4 agonist. Int. Immunopharmacol..

[79] Jin P., Han T.H., Ren J., Saunders S., Wang E., Marincola F.M., Stroncek D.F. (2010). Molecular signatures of maturing dendritic cells: Implications for testing the quality of dendritic cell therapies. J. Transl. Med..

[80] Cruz F.M., Colbert J.D., Merino E., Kriegsman B.A., Rock K.L. (2017). The Biology and Underlying Mechanisms of Cross-Presentation of Exogenous Antigens on MHC-I Molecules. Annu. Rev. Immunol..

[81] Ahmed M.S., Uddin M.J., Hossen M.J., Rahman M.A., Mohibbullah M., Hannan M.A., Choi J.-S. (2022). Dendritic Cells (DCs)-Based Cancer Immunotherapy: A Review on the Prospects of Medicinal Plants and Their Phytochemicals as Potential Pharmacological Modulators. Appl. Sci..

[82] Hostanska K., Hajto T., Spagnoli G.C., Fischer J., Lentzen H., Herrmann R. (1995). A plant lectin derived from Viscum album induces cytokine gene expression and protein production in cultures of human peripheral blood mononuclear cells. Nat. Immun..

[83] Pelzer F., Loef M., Martin D.D., Baumgartner S. (2022). Cancer-related fatigue in patients treated with mistletoe extracts: A systematic review and meta-analysis. Support. Care Cancer.

[84] Schad F., Thronicke A., Hofheinz R.D., Klein R., Grabowski P., Oei S.L., Wüstefeld H., Grah C. (2024). Immune Checkpoint Blockade Combined with AbnobaViscum^®^ Therapy Is Linked to Improved Survival in Advanced or Metastatic Non-Small-Cell Lung Cancer Patients: A Registry Study in Accordance with the ESMO Guidance for Reporting Real-World Evidence. Pharmaceuticals.

[85] Devi S., Gründemann C., Huber R., Kowarschik S. (2023). Characterization of Viscum album L. Effect on Immune Escape Proteins PD-L1, PD-L2, and MHC-I in the Prostate, Colon, Lung, and Breast Cancer Cells. Complement. Med. Res..

[86] Thronicke A., Grabowski P., Roos J., Wüstefeld H., Grah C., Johnson S., Schad F. (2025). Combined Immune Checkpoint Blockade and Helixor^®^ Therapy in Oncology: Real-World Tolerability and Subgroup Survival (ESMO GROW). Int. J. Mol. Sci..

[87] Thronicke A., Reinhold T., von Trott P., Grah C., Matthes B., Matthes H., Schad F. (2020). Cost-effectiveness of real-world administration of chemotherapy and add-on Viscum album L. therapy compared to chemotherapy in the treatment of stage IV NSCLC patients. PLoS ONE.

[88] Wode K., Hök Nordberg J., Kienle G.S., Elander N.O., Bernhardson B.M., Sunde B., Sharp L., Henriksson R., Fransson P. (2020). Efficacy of mistletoe extract as a complement to standard treatment in advanced pancreatic cancer: Study protocol for a multicentre, parallel group, double-blind, randomised, placebo-controlled clinical trial (MISTRAL). Trials.

[89] Oei S.L., Schad F. (2023). Are Aspects of Integrative Concepts Helpful to Improve Pancreatic Cancer Therapy?. Cancers.

[90] Zimmermann-Klemd A.M., Reinhardt J.K., Winker M., Gründemann C. (2022). Phytotherapy in Integrative Oncology-An Update of Promising Treatment Options. Molecules.

[91] Peñaloza E., Holandino C., Scherr C., Araujo P.I.P., Borges R.M., Urech K., Baumgartner S., Garrett R. (2020). Comprehensive Metabolome Analysis of Fermented Aqueous Extracts of *Viscum album* L. by Liquid Chromatography-High Resolution Tandem Mass Spectrometry. Molecules.

[92] Korcan S.E., Çankaya N., Azarkan S.Y., Bulduk İ., Karaaslan E.C., Kargıoğlu M., Konuk M., Güvercin G. (2023). Determination of Antioxidant Activities of *Viscum album* L.: First Report on Interaction of Phenolics with Survivin Protein using in silico Analysis. ChemistrySelect.

[93] Mascher A., Pelzer F., Duncan L.J., Martin D.D., Baumgartner S., Berger B. (2023). The Introspective Patient Experience of Mistletoe Therapy in Cancer: A Qualitative Study. Integr. Cancer Ther..

[94] Guglielmetti G., Baumgartner S., Scherr C., Martin D., Tournier A.L. (2024). Chronobiology of *Viscum album* L.: A time series of daily metabolomic fingerprints spanning 27 years. Front. Physiol..

[95] Acuña C., Kokornaczyk M.O., Baumgartner S., Castelán M. (2023). Unsupervised Deep Learning Approach for Characterizing Fractality in Dried Drop Patterns of Differently Mixed *Viscum album* Preparations. Fractal Fract..

[96] Park J.K., Lee D., Rui L., Gao X., Furness M.S., Wu C. (2025). Analysis of Regulatory Botanical Submission Profile for Cancer Management from the U.S. FDA Perspectives. Ther. Innov. Regul. Sci..

[97] Klingemann H. (2023). *Viscum album* (mistletoe) extract for dogs with cancer?. Front. Vet. Sci..

[98] Biegel U., Mevissen M., Schuller S., Ruess K., Christen O., Ayrle H., Koch C., Walkenhorst M. (2022). *Viscum album* L., a Therapeutic Option for Neoplastic Diseases in Companion Animals? A Systematic Review. Complement. Med. Res..

[99] Wu C., Lee S.L., Taylor C., Li J., Chan Y.M., Agarwal R., Temple R., Throckmorton D., Tyner K. (2020). Scientific and Regulatory Approach to Botanical Drug Development: A U.S. FDA Perspective. J. Nat. Prod..

[100] Gravelin M., Wright J., Holbein M.E.B., Berro M., Brown J.S., Mashour G.A., Weatherwax K.J. (2021). Role of CTSA institutes and academic medical centers in facilitating preapproval access to investigational agents and devices during the COVID-19 pandemic. J. Clin. Transl. Sci..

